# Expanding the Phenotypic Spectrum of *ECEL1*-Associated Distal Arthrogryposis

**DOI:** 10.3390/children8100909

**Published:** 2021-10-13

**Authors:** Akshata Huddar, Kiran Polavarapu, Veeramani Preethish-Kumar, Mainak Bardhan, Gopikrishnan Unnikrishnan, Saraswati Nashi, Seena Vengalil, Priyanka Priyadarshini, Karthik Kulanthaivelu, Gautham Arunachal, Hanns Lochmüller, Atchayaram Nalini

**Affiliations:** 1 Department of Neurology, National Institute of Mental Health and Neuro-Sciences, Bengaluru 560029, India; akshatahuddar@yahoo.com (A.H.); prthshkumar@gmail.com (V.P.-K.); bardhan.mainak@gmail.com (M.B.); gopikrishnanu@gmail.com (G.U.); nandanashi@gmail.com (S.N.); seenavengalil@gmail.com (S.V.); 2 Children’s Hospital of Eastern Ontario Research Institute, Department of Medicine, Division of Neurology, The Ottawa Hospital, Brain and Mind Research Institute, University of Ottawa, Ottawa, ON K1H 8L1, Canada; kpolavarapu@cheo.on.ca (K.P.); hlochmuller@toh.ca (H.L.); 3 Department of Neuro Imaging and Interventional Radiology, National Institute of Mental Health and Neuro-Sciences, Bengaluru 560029, India; priyankapriyadarshini1986@gmail.com (P.P.); pammalkk@gmail.com (K.K.); 4 Department of Human Genetics, National Institute of Mental Health and Neuro-Sciences, Bengaluru 560029, India; gautham.arunachal@gmail.com

**Keywords:** distal arthrogryposis, AMC, *ECEL1*, contractures, muscle MRI

## Abstract

Distal arthrogryposis type 5D (DA5D), a rare autosomal recessive disorder, is caused by mutations in *ECEL1.* We describe two consanguineous families (three patients) with novel *ECEL1* gene mutations detected by next-generation sequencing (NGS). A 12-year-old boy (patient 1) presented with birth asphyxia, motor developmental delay, multiple joint contractures, pes planus, kyphoscoliosis, undescended testis, hypophonic speech with a nasal twang, asymmetric ptosis, facial weakness, absent abductor pollicis brevis, bifacial, and distal lower limb weakness. Muscle MRI revealed asymmetric fatty infiltration of tensor fascia lata, hamstring, lateral compartment of the leg, and gastrocnemius. In addition, 17-year-old monozygotic twins (patients 2 and 3) presented with motor development delay, white hairlock, hypertelorism, tented upper lip, bulbous nose, tongue furrowing, small low set ears, multiple contractures, pes cavus, prominent hyperextensibility at the knee, hypotonia of lower limbs, wasting and weakness of all limbs (distal > proximal), areflexia, and high steppage gait. One had perinatal insult, seizures, mild intellectual disability, unconjugated eye movements, and primary optic atrophy. In the twins, MRI revealed extensive fatty infiltration of the gluteus maximus, quadriceps, hamstrings, and anterior and posterior compartment of the leg. Electrophysiology showed prominent motor axonopathy. NGS revealed rare homozygous missense variants c.602T > C (p.Met201Thr) in patient 1 and c.83C > T (p.Ala28Val) in patients 2 and 3, both localized in exon 2 of *ECEL1* gene. Our three cases expand the clinical, imaging, and molecular spectrum of the *ECEL1*-mutation-related DA5D.

## 1. Introduction

Arthrogryposis multiplex congenita (AMC) is a heterogeneous group of disorders characterized by multiple nonprogressive congenital joint contractures involving at least two different body parts [1]. Distal arthrogryposis (DAs) is diagnosed when contractures mainly involve distal joints of hands, feet, wrist, and ankle [1,2]. DAs are caused by mutations in genes encoding contractile proteins of skeletal myofibers and are further subdivided into 10 different phenotypic and genetic forms caused by *TPM2*, *TNNI2*, *TNNT3*, *MYH3*, *MYBPC1*, *MYH8*, *FBN2*, *PIEZO2*, and *ECEL1* [2,3]. Distal arthrogryposis type 5D (DA5D; OMIM 108145) is described as a rare autosomal recessive DA unlike other dominant forms and has a wide phenotypic spectrum including joint contractures, camptodactyly, hip dislocation, scoliosis, lower limb atrophy, clubfoot, dysmorphic features, furrowed tongue, and asymmetric or unilateral ptosis. Typically, there is normal intelligence and an absence of ophthalmoplegia [3,4]. DA5D is caused due to compound heterozygous or homozygous mutations in membrane-bound metalloprotease endothelin-converting enzyme-like 1 (*ECEL1* (OMIM 605896), also termed DINE in rodents) [3,5]. *ECEL1* is predominantly expressed in neuronal cells and plays an important role in the final axonal arborization of motor nerves to the endplate of skeletal muscles, resulting in the poor formation of the neuromuscular junction [3,5]. We describe two families with three affected individuals with novel *ECEL1* gene mutations with additional features, which thus expands the clinical and imaging spectrum of DA5D.

## 2. Materials and Methods

### 2.1. Patients

The patients were identified and thoroughly investigated with standard clinical and electrophysiological examinations at the specialized neurology and neuromuscular clinic, Department of Neurology, National Institute of Mental Health and Neurosciences, India. Institutional Ethics committee approval (NIMHANS/IEC/2020-21) was obtained to collect all clinical, electrophysiological, and genetic data from the medical records. Patients and parents provided written informed consent to publish the patient’s details, along with face recognition in the clinical photographs and videos. All evaluated patients underwent a thorough clinical examination, and details were recorded in a pre-designed proforma.

### 2.2. Genetic Analysis

The DNA was extracted from blood samples using the QIAamp DNA Blood Mini Kit (QIAGEN, Hilden, Germany). We analyzed both families by Trios next-generation sequencing (NGS) for identification of a genetic cause. Patient 1, along with parents in family 1, underwent whole-exome sequencing (exome research panel by integrated DNA technologies (Coralville, IA, USA) having 39 mb probe span of the human genome and covering coding regions of 19,396 genes) with a mean sequencing coverage of >50–60 X on Illumina (San Diego, CA, USA) sequencing platform. Patients 2 and 3, along with parents in family 2, underwent clinical exome sequencing (custom panel by Agilent technologies (Santa Clara, CA, USA) having 29 mb probe span covering coding regions of 8332 known disease-associated genes) with a mean sequencing coverage of >80–100 X on Illumina sequencing platform. Bioinformatic analysis was concentrated on the analysis of significant variants in 48 known hereditary arthrogryposes and congenital myasthenic syndrome genes for patient 1 and 123 known genes associated with hereditary neuropathies and arthrogryposis/congenital myasthenic syndromes for patients 2 and 3 (Tables S1 and S2). Germ-line variants were identified by aligning the obtained sequences to the human reference genome (GRCh37/hg19) using the BWA program and analyzed using the Genome Analysis Toolkit best-practices variant-calling pipeline [6,7]. The variants were annotated using the Ensemble (release 89) human gene model, with disease annotations ClinVar, SwissVar, and the licensed Human Gene Mutation Database; population frequencies from the 1000 Genome Phase 3, ExAC, gnomAD, and dbSNP databases, and the internal Indian-specific database, as well as in silico prediction algorithms in PolyPhen-2, SIFT, Mutation Taster 2, and LRT. The pathogenicity of the variants was assessed based on 2015 American College of Medical Genetics (ACMG) guidelines [8].

## 3. Results

**Figure 1:**
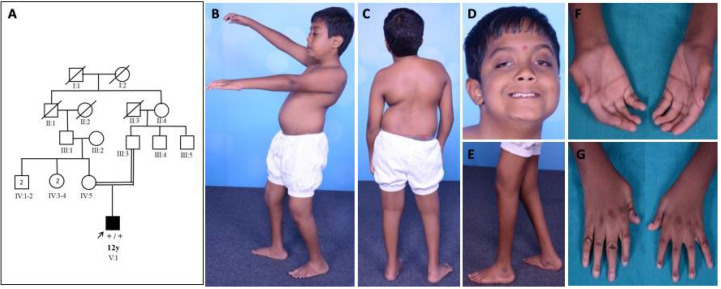
Pedigree and clinical images of patient 1 with DA5D: (**A**) pedigree of family 1; (**B**,**C**) kyphoscoliosis with bilateral hip and knee contracture; (**D**) asymmetric ptosis; (**E**) calf atrophy, contracture at the knee, pes planus, prominent calcaneum; (**F**,**G**) contractures of fingers with absent abductor pollicis brevis.

Family 1: patient 1 was a 12-year-old boy evaluated in the year 2016. He was born to consanguineous parents at term by forceps delivery following an uneventful antenatal period and birth weight of 2.25 kg. There was a history of birth asphyxia (delayed cry at birth and neonatal intensive care unit (NICU) stay for one week). He had foot deformities (pes planus, right eqinovarus) at birth. There was a motor developmental delay, normal mental functions, progressive ptosis, limitation of movement at elbow and hip, and altered gait. He had phimosis, undescended testis, and recurrent urinary tract infection. On examination, he had long eyelashes, low set ears, trismus, high arched palate, asymmetrical ptosis, left eye proptosis with normal fundus, complete extraocular muscle movements, bifacial weakness, hypophonic speech with a nasal twang, taut skin of fingers and face, ulnar deviation of the wrist, absent abductor pollicis brevis, severe contractures of fingers with flexion deformity, contractures at elbows, hip, knee and ankle, pes planus, right side equinovarus deformity, kyphoscoliosis, and calf atrophy with mild distal limb weakness (Medical Research Council grade (MRC) 4) and preserved tendon reflexes (Figure 1).

**Figure 2:**
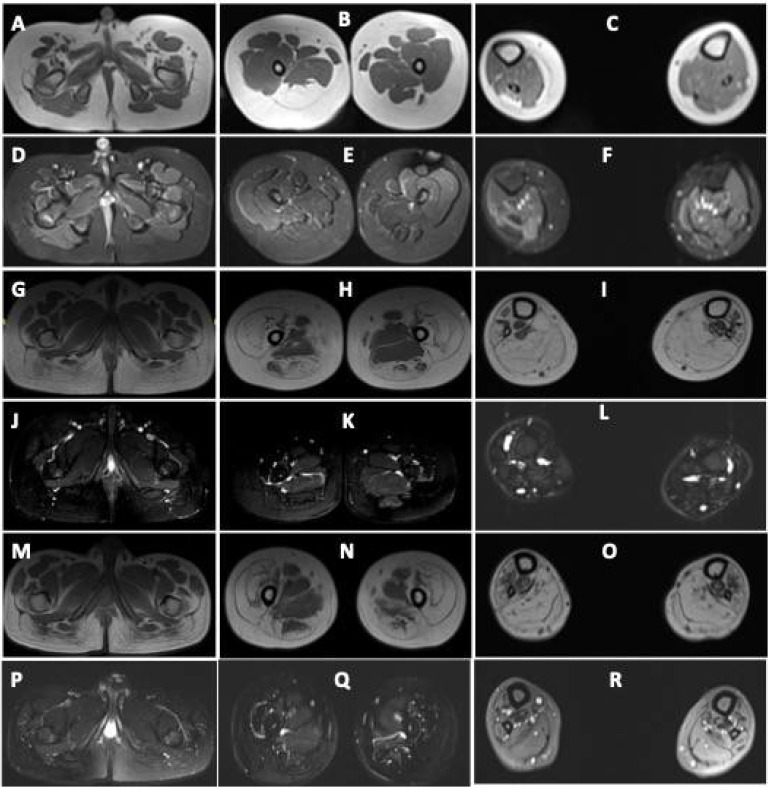
Muscle MRI images of patients with DA5D: (**A**–**F**) muscle MRI images of patient 1: axial T1-weighted sections at the level of the (**A**) pelvis, (**B**) midthigh, and (**C**) midleg reveal volume loss with fatty replacement, most pronounced in bilateral tensor fascia lata (Mercuri Grade 3), right semimembranosus (Grade 3), semitendinosus (Grade 3), bilateral biceps femoris (Grade 2b). Grade 2a fatty replacement is seen in the lateral compartment of the right leg and gastrocnemius. Axial fat-saturated T2-weighted sections at the level of the (**D**) pelvis, (**E**) midthigh, and (**F**) midleg reveal no edema; (**G**–**L**) MRI images of patient 2 and (**M**–**R**) of patient 3: axial T1-weighted sections at the level of the (**G**,**M**) pelvis, (**H**,**N**) midthigh, and (**I**,**O**) midleg reveal volume loss with fatty replacement, most pronounced in bilateral gluteus maximus (Mercuri Grade 3), vasti (Grade3), rectus femoris (Grade 2a), semimembranosus (Grade 2b), semitendinosus (Grade 2b), biceps femoris (Grade 2b). Grade 2b fatty replacement is seen in the anterior, lateral, and deep posterior compartments of the legs. The superficial posterior compartment of both legs reveals Grade 4 atrophy with fatty replacement. Axial fat-saturated T2-weighted sections at the level of the (**J**,**P**) pelvis, (**K**,**Q**) midthigh, and (**L**,**R**) midleg reveal no fluid signal.

He was able to walk independently with a limp and mild waddling. Diagnosis of arthrogryposis multiplex congenita (AMC) was considered. Muscle MRI revealed asymmetric fatty infiltration (right > left) in tensor fascia lata, hamstring, lateral compartment of the right leg, and gastrocnemius (Figure 2). Brain MRI was normal. At the last follow-up (17 years of age) during July 2021, the clinical condition was stationary.

**Figure 3:**
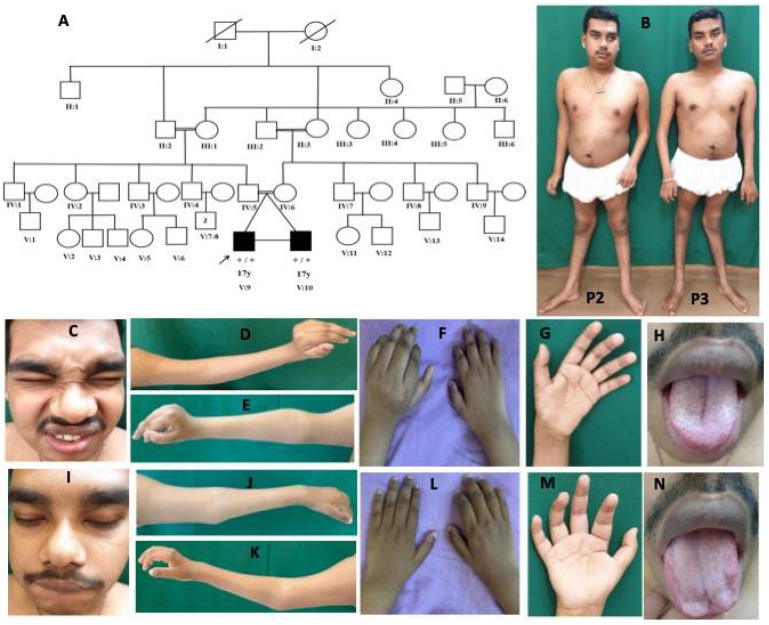
Pedigree and clinical images of patients 2 and 3 with DA5D: (**A**) pedigree of family 2; (**B**) clinical photograph of patients 2 and 3 showing contractures at elbows, fingers, hyperlordosis, and hyperextension at the knee; (**C**–**H**) patient 2: (**C**) facial weakness, hypertelorism, bulbous nose; (**D**–**G**) Left > Right contracture at elbows, wrist in extension and fingers at metacarpophalangeal (MCP) and interphalangeal joint (IPJ), and (**H**) furrowed tongue; (**I**–**N**) patient 3: (**I**) facial weakness, hypertelorism, bulbous nose; (**J**–**M**) Right > Left contracture at elbows, wrist in extension, and fingers at MCP and IPJ, and (**N**) atrophy of tongue.

Family 2: patient 2 was a 17-year-old-boy evaluated during June 2018. He is the first of the twins, born to consanguineous parents at term by normal vaginal delivery following an uneventful antenatal period, and had a birth weight of 1.75 kg (<5th percentile). Fetal movements were normal. He did not cry at birth and had recurrent seizures from day 3 of life and was kept in NICU for 1 week. Subsequently, the child was noticed to have delayed acquisition of all milestones, mild intellectual disability, and recurrent seizures since 8 years of age. He has never been able to walk independently, has altered high stepping gait with slowly progressive weakness of all limbs. Examination revealed flat occiput, hypertelorism, tented upper lip, bushy eyebrows, small low set ears, bulbous nose, a central deep furrow of the tongue, white hairlock, unconjugated eye movements, nonparalytic squint, primary optic atrophy, normal extraocular movements, bifacial weakness, small hands and fingers, asymmetric contractures at fingers (metacarpophalangeal and interphalangeal joints), wrist in extension, and elbow with prominent hyperextensibility at the knees and pes cavus (Figure 3).

There was diffuse atrophy of limb muscles with hypotonia, weakness of all limbs (distal > proximal, MRC grade: shoulder (4), elbow (3), wrist (3), fingers (3), hip (4), knee (4), ankle (0)), and absent tendon reflexes. A clinical diagnosis of hereditary motor neuropathy with unusual contractures was considered. At the last follow-up (20 years of age) during July 2021, seizure frequency had reduced, and the patient needed more support to ambulate.

Patient 3 is the monozygotic twin of patient 2. He was born at term by normal delivery with a birth weight of 1.75 kg (<5th percentile) and had normal perinatal history. He presented with delay in motor milestones, started walking independently at 6 years of age with altered high stepping gait, and had slowly progressive weakness and wasting of limbs. There was no history of seizures or intellectual disability. On examination, he had hypertelorism, tented upper lip, white hairlock, bulbous nose, mild tongue furrowing, normal fundus, and extraocular movements, mild bifacial weakness, asymmetric contractures at fingers (metacarpophalangeal and interphalangeal joints), wrist in extension, and elbow with knee hyperextensibility and pes cavus (Figure 3). There was diffuse atrophy of limb muscles with hypotonia, weakness of limbs (distal > proximal MRC grade: shoulder (4), elbow (4), wrist (4), fingers (3), hip (4), knee (3), ankle (3)), and absent tendon reflexes. At the last follow-up (20 years of age) during July 2021, the clinical condition was stationary.

Investigations in the twins revealed normal creatine kinase, hepatic, renal, and thyroid function tests. Serum lactate was 41.2 (patient 2) and 26.3 (patient 3) (reference range: 4.5 to 20 mg/dl), while ammonia, HbA1c, homocysteine, plasma amino acids, and acylcarnitine profile were normal in both. Nerve conduction studies (NCS) in both revealed normal sensory nerve conductions (right median, ulnar and sural), impaired motor conduction studies of the right median, and ulnar with reduced amplitude, normal latency, and conduction velocities (Table 1). The right common peroneal nerve was inexcitable. EMG of the tibialis anterior showed fibrillations, positive sharp waves with high amplitude polyphasic motor unit action potentials (MUAPs) with mildly reduced recruitment. Similarly, abductor digiti minimi (ADM) showed evidence of high amplitude polyphasic MUAPs, suggestive of a neurogenic pattern. Repetitive nerve stimulation at 3Hz did not reveal a significant decrement response from orbicularis oculi, trapezius, and ADM muscles.

Muscle MRI in both revealed volume loss with fatty replacement, most pronounced in bilateral gluteus maximus, vasti, rectus femoris, hamstrings, and all muscles of the anterior, lateral, and posterior compartments of legs. Hip adductors and sartorius were spared (Figure 2).

Brain MRI in patient 2 revealed focal encephalomalacia with adjacent gliosis in bilateral parieto-occipital regions with a paucity of white matter, thinning of body, and splenium of corpus callosum suggestive of hypoxic-ischemic injury. Brain MRI in patient 3 revealed symmetric T2/FLAIR hyperintensities in bilateral parieto-occipital and corticospinal tracts.

### Genetic Results

Trios NGS performed in both families identified *ECEL1* homozygous disease-causing variants in patients 1, 2, and 3 from families 1 and 2, respectively. Patient 1 had a novel homozygous missense variant in exon 2 of *ECEL1* (NM_004826.4): c.602T > C (p.Met201Thr), in the extracellular Peptidase M13 domain (Uniprot). The variant is present in heterozygous form in both parents and is not reported in the general population (Gnomad frequency: 0). In silico predictions determined the variant as damaging/pathogenic, and it is classified as “likely pathogenic” as per ACMG criteria (PM1, PM2, PP2, PP3).

Patients 2 and 3 were identified to have a novel homozygous missense variant in exon 2 of *ECEL1*: c.83C > T (p.Ala28Val), which is located in the proximal cytoplasmic domain. While the variant is segregated as heterozygous in both parents, it is present with low frequency in the general population (Gnomad MAF: 0.005%; Heterozygotes −3; Homozygotes-nil). The in silico predictions were pathogenic by SIFT and additional analysis by human splicing finder (HSF—https://hsf.genomnis.com/home, accessed on 15 April 2021) [9] showed “Potential alteration of splicing due to activation of a cryptic donor site”. Based on ACMG criteria, c.83C > T (p.Ala28Val) has been classified as “likely pathogenic” (PM1, PM2, PP2, PP3).

## 4. Discussion

Arthrogryposis multiplex congenita is a heterogeneous condition caused by a myriad of disorders including aneuploidy syndromes, skeletal dysplasias, multiple congenital anomaly syndromes, and neuromuscular diseases [1,2]. Among these, a group of disorders characterized mainly, but not exclusively, by abnormalities of the distal limbs were described as distal arthrogryposes (DAs) in 1982, by Hall et al. [2]. Subsequently, DA has been defined as an inherited primary limb malformation disorder characterized by congenital contractures of two or more different body areas and without primary neurologic and/or muscle disease that affects limb function [2]. Major diagnostic criteria include ulnar deviation, camptodactyly (or pseudocamptodactyly), hypoplastic and/or absent flexion creases, and or overriding fingers, talipes equinovarus, calcaneo-valgus deformities, vertical talus, and/or metatarsus varus [2].

Most DAs are autosomal dominant disorders caused by genes encoding proteins related to the muscle contraction apparatus [2,3]. However, distal arthrogryposis type 5D is autosomal recessive and is usually caused by biallelic mutations in *ECEL1* [3].

Distal arthrogryposis type 5 D is characterized by a wide array of clinical features including (i) musculoskeletal with foot deformities, finger contractures, and limited movement of proximal joints, recurrent hip dislocation, webbing of fingers and neck, scoliosis, kyphosis, muscle atrophy, and weakness; (ii) ophthalmological with asymmetric ptosis, strabismus, refractive errors, and ophthalmoplegia; (iii) facial with arched eyebrows, bulbous upturned nose, micrognathia, small mouth, reduced facial expression, cleft palate and tongue atrophy; (iv) others including speech difficulties, nasal voice, short stature, short neck, cryptorchidism, pterygia, faint palmar creases, and respiratory dysfunction [3,4,5,10,11,12,13,14,15,16,17,18,19]. Progressive scoliosis and weakness of limbs have been reported on long-term follow-up of these patients [11]. Consanguineous parentage with a history of reduced fetal movements may give an additional clue to the diagnosis. The fetal movements were normal in our patients. Characteristics of patients reported in the literature and our patients with DA5D are summarized in Table 2.

In addition to features described earlier, our patients had additional features of white hairlock, proptosis, prominent knee hyperextensibility, and areflexia, thus further expanding the clinical spectrum of this disorder. However, distal interphalangeal joint hyperlaxity and areflexia have been reported in a few patients [4]. Global development delay, recurrent seizures, and mental subnormality in patient 2 can possibly be attributed to birth asphyxia and brain injury.

*ECEL1* encodes endothelin-converting enzyme-like 1, a type II integral transmembrane zinc metalloprotease, similar to the endothelin-converting enzyme (ECE) structurally but functionally different, as *ECEL1* does not cleave ECE substrates [5,10]. Mouse studies have shown that damage-induced neuronal endopeptidase (DINE; rodent homolog of *ECEL1*) is significantly upregulated in both the peripheral and central nervous systems. *ECEL1* is essential for the final axonal arborization of motor nerves in the diaphragm, limb skeletal muscles, and for the formation of proper neuromuscular junctions (NMJs) during prenatal development [10]. Failure of formation and maturation of the embryonic neuromuscular end plate and NMJs leads to early and sustained lack of movement in utero causing pterygia, webs, and contractures [10]. Further, the twins had prominent foot drop mimicking a progressive motor neuropathy, which was also corroborated the severe motor axonopathy. These features have not been reported earlier in English literature.

**Figure 4:**
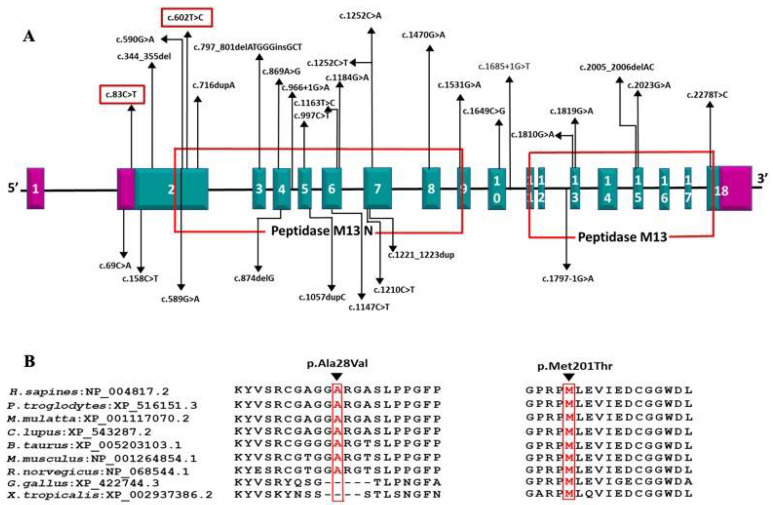
(**A**) Schematic representation of the variations identified in the *ECEL1* gene with corresponding exons and protein domains. The 18 exons of *ECEL1* (NM_004826.4) are represented as boxes with respective exon numbers. Regions of exons coding for UTRs are marked in violet color at the ends. The protein domains are according to the Uniprot database. The novel variant found in our is study is marked in the box. The size of exons /introns is not represented at scale; (**B**) amino acid conservation at positions 28 and 201 across species.

DA5D is caused by recessive mutations in the *ECEL1* gene [3,4]. We identified homozygous novel missense variants c.602T > C (p.Met201Thr) and c.83C > T (p.Ala28Val) in exon 2 of *ECEL1* gene in families 1 and 2, respectively. The c.602T > C (p.Met201Thr) variant affects the crucial Peptidase M13 extracellular domain where all the previous disease-causing missense mutations have been reported. Two missense mutations affecting a nearby codon 197 (p.Gly197Asp and p.Gly197Ser) have been reported in DA5D patients of European ancestry by McMillin et al. and Ullmann et al., respectively [3,11] (Figure 4).

These patients had a similar phenotype of fixed contractures at birth and progressive weakness. However, Ullmann et al. reported a consanguineous family with two affected siblings and another affected cousin who had identical p.Gly197Ser homozygous mutation with long-term follow up and disease progression [11]. The elder female sibling in the family did not have ptosis and had additional temporomandibular contractures not reported in other patients [11]. Interestingly, our patient 1 also had trismus due to temporomandibular joint contractures. Ullmann et al. also reported that ambulation is preserved in patients even into the third decade with slowly progressive muscle weakness [11]. Mild learning difficulties and exercise intolerance were some of the unusual findings identified in the elder sibling on long-term follow-up [11]. However, brain MRI was not performed, and there was no evidence of NMJ dysfunction in EMG study [11]. Likewise, our patients 1 and 3 had a stable nonprogressive course and were independently ambulant at 15 and 20 years, respectively. However, patient 3 had a significant motor disability and remains status quo. 

In patients 2 and 3 (twin siblings affected in family 2), the c.83C > T (p.Ala28Val) variant was identified in the proximal cytoplasmic domain. Based on literature evidence and ClinVar-reported mutations, only two nonsense mutations c.69C > A (p.Cys23Ter) and c.33C > G (p.Tyr11Ter) associated with DA5D phenotype have been reported previously in the cytoplasmic domain of *ECEL1* [10,20]. The phenotypic pattern of fixed contractures at birth with slowly progressive weakness reported with proximal nonsense mutations was not dissimilar to those with mutations in the downstream extracellular domain, suggesting a common pathomechanism irrespective of location and type of mutation. The mutation c.83C > T (p.Ala28Val) identified in our study is the first disease-causing missense variant affecting the proximal cytoplasmic domain of *ECEL1*. Additional in silico analysis by human splicing finder (HSF) predicted that c.83C > T can cause significant alteration of wild type splicing mechanism by activating a cryptic donor splice site in exon 2 and also altering the ratio of exonic-splicing enhancers and silencers (ESE/ESS). While this can result in partial deletion of exon 2 and/or exon skipping, additional RNA analysis might be required to confirm the impact on protein expression [9]. We admit that the lack of functional validation for c.83C > T (p.Ala28Val) is a limitation for this study due to the nonavailability of tissue samples from patients. Nevertheless, these in silico predictions suggest a loss of function mechanism similar to previous nonsense mutations identified in the proximal cytoplasmic domain [10,20].

There are only a few reports on muscle MRI in patients with DA5D [4,11]. Severe fatty infiltration of thighs affecting the biceps femoris, sartorius and vastus lateralis, extensor digitorum longus, and asymmetric involvement of distal leg muscles with sparing of rectus femoris and gracilis has been reported [4,11]. All three patients in the current study underwent muscle MRI. Diffuse fatty infiltration was observed involving hamstrings and gastrocnemius in all, and extensive fatty infiltration of gluteus maximus, quadriceps, anterior, lateral, and posterior compartments of legs was also observed in the two monozygotic twins. This further implies a wider spectrum of disease involvement. It is interesting to note that the severity of muscle weakness was different in patients 2 and 3, but the severity of MRI findings was almost identical.

## 5. Conclusions

Distal arthrogryposis type 5D is a very rare autosomal recessive disorder caused by mutations in *ECEL1* characterized predominantly by distal contractures. Being an autosomal recessive disorder, it has implications in genetic counseling. Here, we described three patients with novel mutations, and additional clinical and imaging features compared with earlier descriptions, thus expanding the clinical, imaging, and molecular spectrum of the *ECEL1* mutations and associated DA5D.

## Figures and Tables

**Table 1:** Nerve conduction studies of patients 2 and 3.

Parameters		Patient 2	Patient 3
**Motor conduction study**	Distal Latency(ms)/CMAP(mV)/MNCV (m/s)		
	Median	3.84/1.73/49.3	3.5/1.32/60.6
	Ulnar	3.86/0.8/49.7	2.25/2.61/61.8
	CPN	absent	absent
**Sensory conduction study**	Onset Latency(ms)/SNAP(uV)/SNCV (m/s)		
	Median	3.04/12.8/52.6	2.86/20.1/55.9
	Ulnar	2.84/10.76/50	2.12/15.4/56.9
	Sural	3.02/8.79/53	3.06/11/45.8

ADM: abductor digiti minimi, CMAP: compound motor action potential, CPN: common peroneal nerve, MNCV: motor nerve conduction velocity, NA: not available, SNCV: sensory nerve conduction velocity.

**Table 2:** Comparison of clinical features of this study with previous studies.

Clinical Features	McMillin et al.	Dieterich et al.	Shaheen et al.	Shaaban et al.	Patil et al.	Barnett et al.	Bayram et al.	Hamzeh et al.	Ullmann et al.	Stattin et al.	Umair et al.	Jin et al.	Alei et al.	Total	Present Study
Year of study	2013	2013	2014	2014	2014	2014	2016	2017	2018	2018	2019	2020	2021		2021
Number of patients	9	10	9	2	1	2	4	1	7	1	2	1	2	51	3
Consanguinity	2/9	9/10	9/9	2/2	1/1	0/2	4/4	1/1	4/7	1/1	2/2	0/1	1/2	36/51	3/3
Male:Female	5:4	5:5	3:6	1:1	0:1	1:1	3:1	1:0	2:5	1:0	2:0	1:0	0:2	25:26	3:0
Contractures															
Foot or toe contractures and/or deformity	9/9	9/10	4/9	2/2	1/1	2/2	3/3	1/1	6/7	1/1	2/2	0/1	2/2	42/50	3/3
Ankle	9/9	NA	NA	0/2	NA	2/2	NA	NA	6/7	1/1	2/2	NA	NA	20/23	1/3
Knee	8/9	10/10	5/5	2/2	1/1	2/2	1/1	1/1	7/7	1/1	2/2	1/1	2/2	43/44	1/3
Hip dislocation and/or limitation of movement	9/9	9/9	6/9	0/2	1/1	2/2	3/3	1/1	5/7	1/1	NA	NA	1/2	38/46	1/3
Hand and/or finger	9/9	10/10	9/9	2/2	1/1	2/2	4/4	1/1	7/7	1/1	2/2	1/1	2/2	51/51	3/3
Wrist	9/9	NA	1/1	2/2	NA	2/2	NA	NA	2/7	1/1	NA	1/1	2/2	20/25	2/3
Elbow	5/5	3/7	1/1	0/2	1/1	2/2	1/1	1/1	3/7	1/1	2/2	1/1	1/2	22/33	3/3
Shoulder	6/6	2/8	1/1	1/2	0/1	0/2	NA	1/1	4/7	1/1	NA	NA	2/2	18/31	0/3
Neck	4/4	NA	NA	2/2	NA	1/2	NA	NA	NA	NA	NA	NA	2/2	9/10	0/3
Webbed neck	3/8	NA	NA	2/2	NA	NA	NA	1/1	2/7	1/1	0/2	NA	NA	9/21	0/3
Ptosis	8/9	7/10	6/9	½	1/1	2/2	1/1	1/1	5/7	1/1	2/2	1/1	1/2	37/48	1/3
Strabismus	1/1	1/10	3/9	2/2	0/1	NA	NA	NA	1/7	NA	2/2	1/1	1/2	12/35	1/3
Ophthalmoplegia	0/9	1/10	NA	2/2	0/1	NA	NA	NA	0/7	NA	0/2	NA	1/2	4/33	0/3
Bulbous nose	9/9	NA	2/2	2/2	1/1	NA	2/2	NA	NA	1/1	NA	NA	NA	17/17	2/3
Reduced facial movements	1/9	3/7	NA	2/2	NA	NA	NA	NA	5/7	1/1	NA	1/1	1/2	14/29	3/3
Micrognathia/small mouth	8/9	3/10	1/1	2/2	1/1	1/1	2/2	1/1	4/7	1/1	0/2	NA	2/2	26/39	1/3
Cleft palate	1/1	1/1	NA	NA	1/1	NA	NA	NA	2/7	1/1	0/2	1/1	0/2	7/16	0/3
Tongue atrophy/furrowing	NA	7/7	NA	NA	1/1	0/2	NA	1/1	4/5	1/1	0/2	NA	1/2	15/21	2/3
Short neck	4/7	10/10	NA	2/2	1/1	NA	NA	NA	NA	NA	NA	NA	2/2	19/22	0/3
Speech Abnormalities	NA	5/5	NA	NA	NA	NA	NA	NA	NA	NA	2/2	NA	1/2	8/9	1/3
Scoliosis	2/9	7/10	2/9	2/2	1/1	NA	1/1	1/1	3/7	NA	0/2	NA	2/2	21/44	1/3
Hyperlordosis	NA	9/9	NA	1/2	0/1	NA	NA	NA	NA	NA	NA	NA	2/2	12/14	0/3
Muscle atrophy	NA	10/10	NA	2/2	1/1	NA	NA	1/1	4/7	1/1	1/2	NA	1/2	21/26	3/3

## Data Availability

The clinical and raw genetic data are available for review with the corresponding author and available on request.

## Supplementary Materials

The following are available online at https://www.mdpi.com/article/10.3390/children8100909/s1, Table S1: Coverage of arthrogryposis and congenital myasthenic syndrome panel genes in patient 1, Table S2: Coverage of Charcot-Marie-Tooth and other sensory neuropathies and arthrogryposis & congenital myasthenic syndrome genes in patient 2 and 3.
