# Supplementary material for: Expanding the Phenotypic Spectrum of ECEL1-Associated Distal Arthrogryposis
Source: Children (Basel). 2021 Oct 13;8(10):909. doi: 10.3390/children8100909 (PMC8534696; doi:10.3390/children8100909)
Supplement: Supplementary file 1 [file children-08-00909-s001.zip › Table S1.pdf]

Table S1: Coverage of arthrogryposis and congenital myasthenic syndrome panel genes in patient 1.

Gene	Percentage of coding region covered	Gene	Percentage of coding region covered	Gene	Percentage of coding region covered
ADCY6	100.00	AGRN	100.00	ALG14	100.00
ALG2	100.00	CHAT	100.00	CHRNA1	100.00
CHRNB1	100.00	CHRND	100.00	CHRNE	100.00
CHRNG	100.00	CHST14	100.00	CNTNAP1	100.00
COL13A1	99.27	COLQ	91.25	CORIN	100.00
DNM2	99.57	DOK7	100.00	DPAGT1	100.00
ECEL1	100.00	ERBB3	100.00	FBN2	99.20
GFPT1	100.00	GLE1	100.00	GPR126	100.00
LAMB2	100.00	LAMC1	100.00	LRP4	100.00
MUSK	97.34	MYBPC1	100.00	MYH3	100.00
MYH8	100.00	NALCN	95.42	PI4KA	100.00
PIEZO2	98.91	PIP5K1C	100.00	RAPSN	99.94
SCN4A	100.00	SLC18A3	100.00	SLC35A3	100.00
SNAP25	100.00	SYT2	100.00	TNNI2	100.00
TNNT3	100.00	TPM2	100.00	UBA1	97.08
VIPAS39	100.00	VPS33B	100.00	ZBTB42	100.00
