# Supplementary material for: Expanding the Phenotypic Spectrum of ECEL1-Associated Distal Arthrogryposis
Source: Children (Basel). 2021 Oct 13;8(10):909. doi: 10.3390/children8100909 (PMC8534696; doi:10.3390/children8100909)
Supplement: Supplementary file 1 [file children-08-00909-s001.zip › Table S2.pdf]

Table S2: Coverage of Charcot-Marie-Tooth and other sensory neuropathies and arthrogryposis & congenital myasthenic syndrome genes in patient 2 and 3.

Gene	Percentage of coding region covered	Gene	Percentage of coding region covered	Gene	Percentage of coding region covered
AARS	100.00	*ADCY6*	100.00	*ADGRG6*	100.00
AGRN	100.00	*AIFM1*	100.00	*ALG14*	100.00
ALG2	100.00	*ATL1*	100.00	*ATL3*	100.00
CCT5	100.00	*CHAT*	100.00	*CHRNA1*	100.00
CHRNB1	100.00	*CHRND*	100.00	*CHRNE*	100.00
CHRNG	100.00	*CHST14*	100.00	*CNTNAP1*	100.00
COL13A1	100.00	*COLQ*	100.00	*COX6A1*	100.00
CTDP1	100.00	*DHTKD1*	100.00	*DNAJB2*	100.00
DNM2	100.00	*DNMT1*	100.00	*DOK7*	100.00
DPAGT1	100.00	*DST*	100.00	*DYNC1H1*	100.00
ECEL1	100.00	*EGR2*	100.00	*ERBB3*	100.00
FAM134B	100.00	*FBN2*	100.00	*FGD4*	100.00
FIG4	100.00	*GAN*	100.00	*GARS*	100.00
GDAP1	100.00	*GFPT1*	100.00	*GJB1*	100.00
GLE1	100.00	*GNB4*	100.00	*HARS*	100.00
HINT1	100.00	*HK1*	97.01	*HOXD10*	100.00
HSPB1	100.00	*HSPB8*	100.00	*IGHMBP2*	100.00
INF2	100.00	*JPH1*	100.00	*KARS*	100.00
KIF1A	100.00	*KIF1B*	100.00	*KIF5A*	100.00
LAMB2	100.00	*LITAF*	90.52	*LMNA*	100.00
LRP4	100.00	*LRSAM1*	100.00	*MARS*	100.00
MED25	100.00	*MFN2*	100.00	*MME*	98.13
MORC2	100.00	*MPZ*	100.00	*MTMR2*	100.00
MUSK	100.00	*MYBPC1*	100.00	*MYH3*	100.00
MYH8	100.00	*NAGLU*	100.00	*NALCN*	100.00
NDRG1	100.00	*NEFH*	92.43	*NEFL*	100.00
NGF	100.00	*NTRK1*	100.00	*PDK3*	100.00
PI4KA	100.00	*PIEZO2*	100.00	*PIP5K1C*	100.00
PLEKHG5	100.00	*PMP22*	100.00	*PRDM12*	100.00
PRPS1	100.00	*PRX*	100.00	*RAB7A*	100.00
RAPSN	100.00	*REEP1*	100.00	*SBF1*	100.00
SBF2	100.00	*SCN11A*	100.00	*SCN4A*	100.00
SCN9A	100.00	*SH3TC2*	100.00	*SLC12A6*	100.00
SLC18A3	100.00	*SLC25A46*	100.00	*SLC35A3*	100.00
SNAP25	100.00	*SOX10*	100.00	*SPG11*	100.00
SPTLC1	100.00	*SPTLC2*	100.00	*SURF1*	100.00

SYT2	100.00	*TFG*	100.00	*TNNI2*	100.00
TNNT3	100.00	*TPM2*	100.00	*TRIM2*	100.00
TRPV4	100.00	*TTR*	100.00	*UBA1*	100.00
VCP	100.00	*VIPAS39*	100.00	*VPS33B*	100.00
WNK1	100.00	*YARS*	100.00	*ZBTB42*	100.00
